# Identification of m7G-associated lncRNA prognostic signature for predicting the immune status in cutaneous melanoma

**DOI:** 10.18632/aging.204151

**Published:** 2022-06-29

**Authors:** Jielin Rong, Hui Wang, Yi Yao, Zhengyuan Wu, Leilei Chen, Chaojie Jin, Zhaoyang Shi, Cheng Wu, Xueqing Hu

**Affiliations:** 1 Department of Hand Plastic Surgery, The First People's Hospital of Linping District, Hangzhou 311199, China; 2 Department of Plastic Surgery, The Second Affiliated Hospital of Zhejiang University, Hangzhou 311199, China

**Keywords:** cutaneous melanoma, m7G, lncRNA, gene signature, immune status

## Abstract

RNA modifications, including RNA methylation, are widely existed in cutaneous melanoma (CM). Among epigenetic modifications, N7-methylguanosine (m7G) is a kind of modification at 5’ cap of RNA which participate in maintaining the stability of mRNA and various cell biological processes. However, there is still no study concerning the relationship between CM and m7G methylation complexes, METTL1 and WDR4. Here, long non-coding RNA (lncRNAs) and gene expression data of CM from the Cancer Genome Atlas (TCGA) database were retrieved to identify differentially expressed m7G-related lncRNAs connected with overall survival of CM. Then, Cox regression analyses was applied to construct a lncRNA risk signature, the prognostic value of identified signature was further evaluated. As a result, 6 m7G-associated lncRNAs that were significantly related to CM prognosis were incorporated into our prognostic signature. The functional analyses indicated that the prognostic model was correlated with patient survival, cancer metastasis, and growth. Meanwhile, its diagnostic accuracy was better than conventional clinicopathological characteristics. The pathway enrichment analysis showed that the risk model was enriched in several immunity-associated pathways. Moreover, the signature model was significantly connected with the immune subtypes, infiltration of immune cells, immune microenvironment, as well as several m6A-related genes and tumor stem cells. Finally, a nomogram based on the calculated risk score was established. Overall, a risk signature based on 6 m7G-associated lncRNAs was generated which presented predictive value for the prognosis of CM patients and can be further used in the development of novel therapeutic strategies for CM.

## INTRODUCTION

Cutaneous melanoma (CM) is an aggressive malignant tumor that threatens human life [1]. The pathogenesis and development of CM are negatively correlated with skin pigmentation. Most cases comprise patients with low skin pigmentation and who were exposed to ultraviolet radiation [2]. However, the precise pathogenic mechanisms behind CM remain unknown. In 2018, a total of 287,723 people were diagnosed with melanoma worldwide, and 60,709 died due to this disease [3]. The 10-year overall survival (OS) rates of stages I and II CM patients are 75 to 98% [4]. On the other hand, only 24–88% of CM patients in stages IIIA to IIID survived after 10 years, suggesting that the early diagnosis and treatment of CM might affect its outcome. Hence, accurate diagnosis in relatively early stages can significantly influence CM therapies. Recently, many investigators have attempted to identify novel biomarkers that can be used for prognostic prediction and personalized therapy of CM patients, but only a few biomarkers of clinical significance were identified [5]. Therefore, the identification of new biomarkers that can accurately predict the prognosis of CM patients is urgently needed.

Recently, RNA modification was identified to be connected with a variety of cancers and human physiologies, especially tumor immunity [6, 7]. Among the identified epigenetic modifications which include N1-methyladenosine (m1A), N7-methylguanosine (m7G), and N6-methyladenosine (m6A), m7G is most abundantly medicated at 5′ cap of tRNA, mRNA, microRNAs, and rRNA [8]. This methylation includes two types of complexes, METTL1 and WDR4. In addition to maintain the stability of mRNA [9, 10], m7G modification also participate in pre-mRNA splicing, transcription elongation, and mRNA translation [8, 11]. Meanwhile, abnormal m7G methylation will lead to cancer progression [12]. Nevertheless, little literatures reported the correlation between m7G and cancer prognosis, and the role of m7G methylation in melanoma is still unknown.

Long non-coding RNAs (lncRNAs) are a subset of non-coding RNAs longer than 200 base pairs. In addition to various cellular biological processes, lncRNAs contribute to tumor progression, including tumorigenesis, cell proliferation, and tumor metastasis [13, 14]. In melanoma, various lncRNAs are proved significantly associated with patients’ overall survival [15]. Experimental studies have also confirmed that lncRNAs in melanoma cells contribute to cell cycle progression, apoptosis, cancer invasion, and migration. Furthermore, lncRNAs influence the chemotherapeutic sensitivity of melanoma cells [16]. Based on these studies, lncRNAs are key determinants of prognosis in CM. However, systematic analyses aimed at the identification of hub m7G-associated lncRNAs associated with prognosis or progression are lacking.

Through bioinformatic analysis, many disease-specific biomarkers have been identified. However, m7G-associated lncRNAs related to melanoma progression or prognosis were not previously identified. Therefore, we conducted the analysis of differential gene expression and univariate Cox regression in this study and identified the lncRNAs differentially expressed and correlated with the prognosis of CM patients. Then, hub m7G-associated lncRNAs were characterized, and a gene risk model was constructed using the Lasso penalized Cox regression analysis. The prognostic value and clinical significance of this model were further validated in CM patients. Moreover, we analyzed the connections between the constructed lncRNAs signature and immune infiltrates, immune microenvironment, relationship with m6A genes, and tumor stemness. Currently, risk signature generated according to the expression of m7G-associated lncRNAs in cancer has not been performed yet. Thus, to the best of our knowledge, our present study demonstrated the first m7G-associated lncRNAs for the prediction of cancer prognosis, providing novel insights into the prognosis and diagnosis of CM.

## RESULTS

### Candidate prognostic lncRNA screening

**Figure 1:**
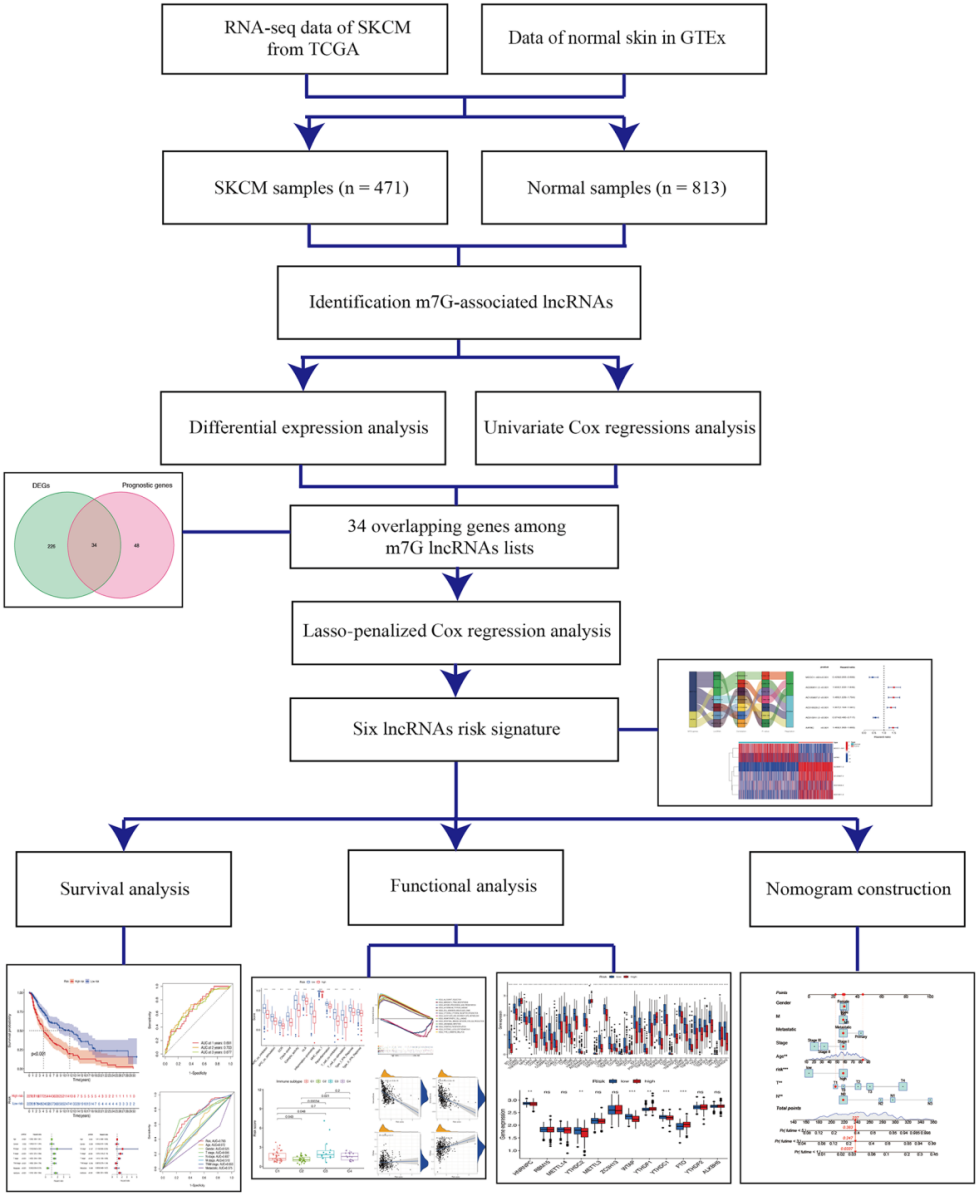
Schema of the study.

**Figure 2:**
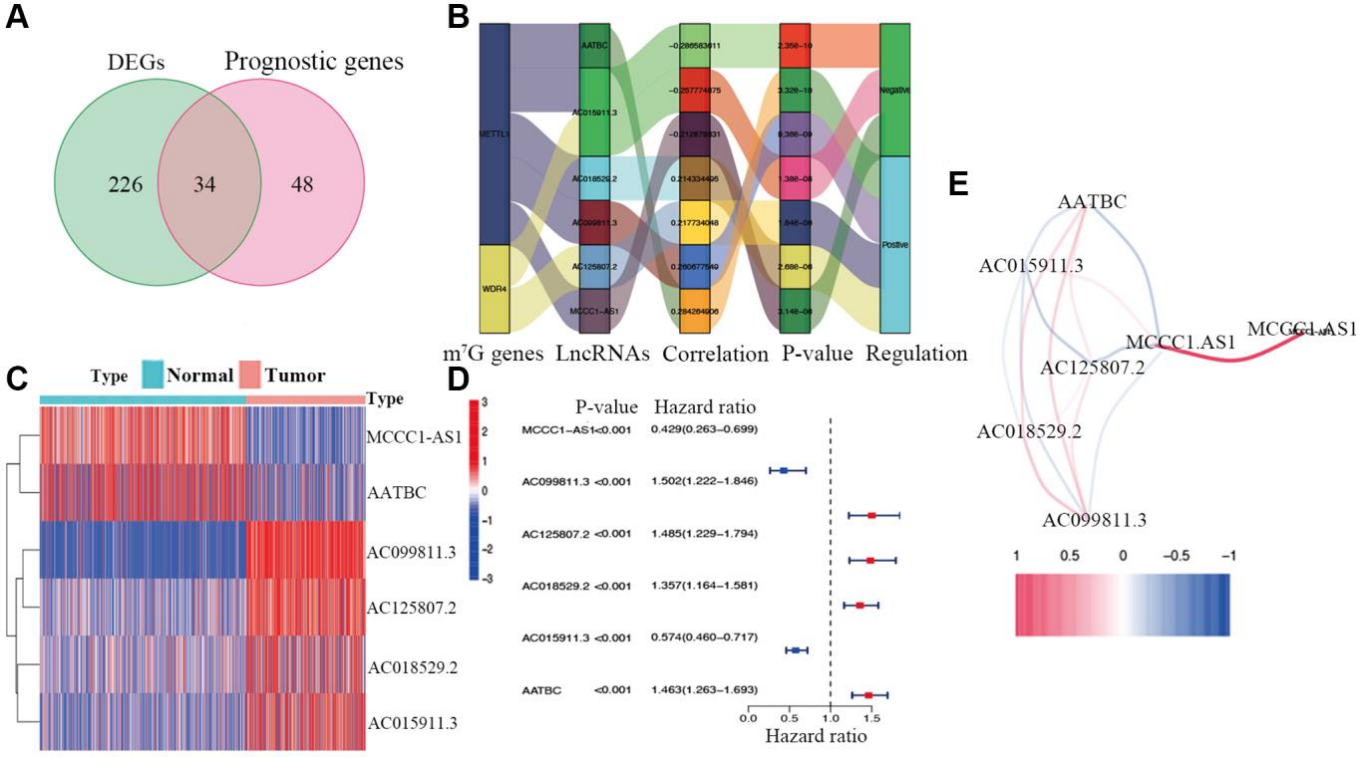
**Identification of prognostic m7G-related lncRNAs.** (**A**) Venn diagram of candidate m7G-related lncRNAs determined by differential expression and univariate Cox analyses. (**B**) Correlation network of prognostic lncRNAs and their associated mRNAs. (**C**) Heatmap of hub m7G-related lncRNAs. (**D**) Forest plots of correlations between hub lncRNAs and overall survival of CM patients. (**E**) Correlation network of hub lncRNAs.

The study workflow is illustrated in Figure 1. First, 638 lncRNAs associated with METTL1 and WDR4 expression in CM patients were identified. The correlations between levels of these lncRNAs and m7G methylation genes are summarized in Supplementary Table 1. Based on a differential expression analysis and univariate Cox regression, 260 and 82 lncRNAs were identified as differentially expressed lncRNAs and prognostic lncRNAs, respectively (Supplementary Tables 2 and 3). Based on the overlap, 34 lncRNAs were identified as candidate prognostic lncRNAs (Figure 2A).

### Construction of a lncRNA signature for CM

**Figure 3:**
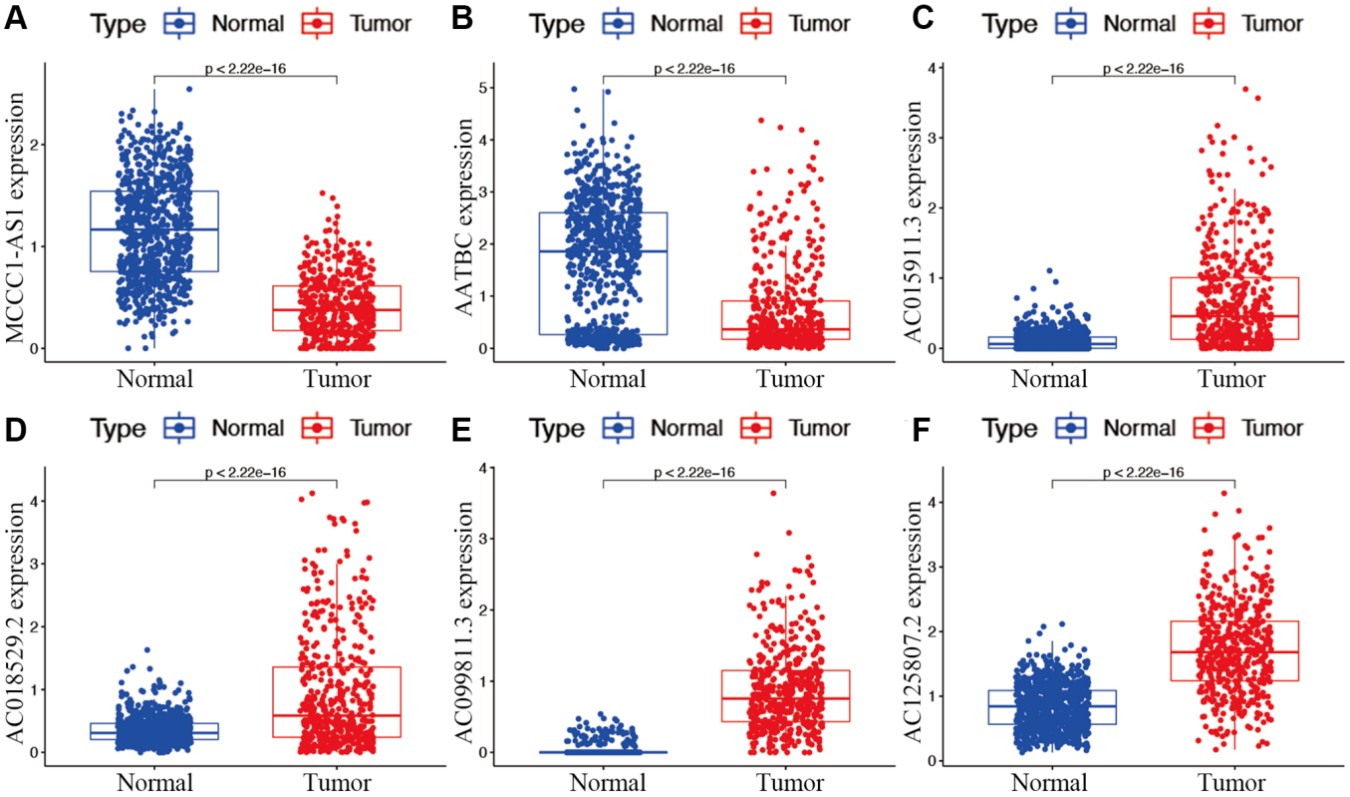
Expression of hub m7G-associated lncRNAs MCCC1-AS1 (**A**), AATBC (**B**), AC015911.3 (**C**), AC018529.2 (**D**), AC099811.3 (**E**), and AC125807.2 (**F**) in risk subgroups.

**Figure 4:**
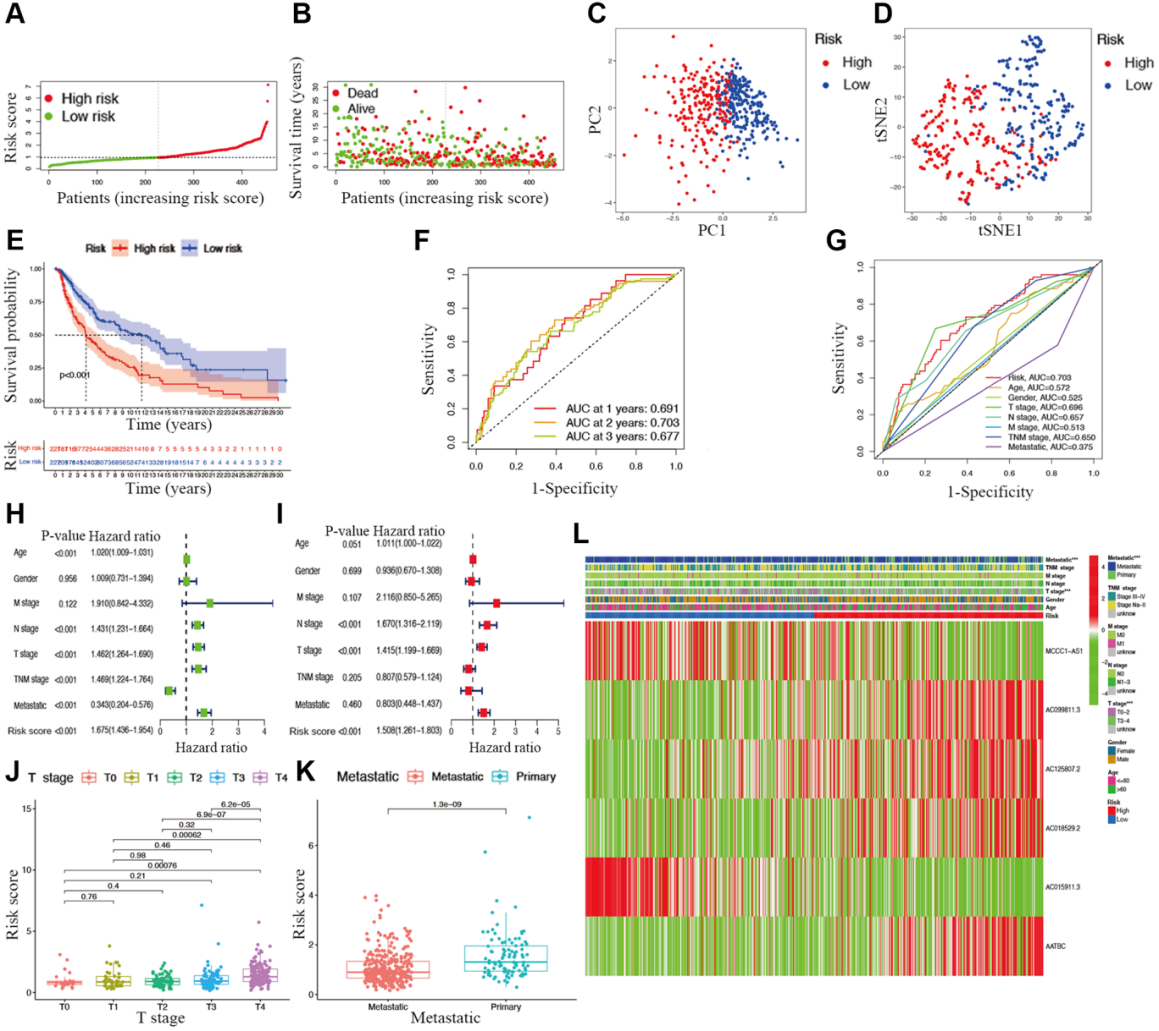
**Associations between risk signature and clinicopathological factors.** Risk score distribution (**A**), survival status (**B**), PCA plot (**C**), and t-SNE (**D**) analysis of TCGA-CM cohort. (**E**) Survival curve of CM patients. TimeROC (**F**) and ClinicalROC (**G**) curves to forecast overall survival of patients. Univariate (**H**) and multivariate Cox (**I**) regression of clinicopathological features in TCGA-CM cohort. Correlations between risk scores and T stage (**J**) and metastatic capacity (**K**). (**L**) The heatmap of clinicopathological features and hub lncRNAs expression in two risk subgroups.

Candidate prognostic lncRNAs were further analyzed by a Lasso penalized Cox regression analysis, and 6 hub lncRNAs (MCCC1-AS1, AC099811.3, AC125807.2, AC018529.2, AC015911.3, AATBC) were ultimately applied to construct the risk signature (Supplementary Table 4). The connection between the identified hub lncRNAs, METTL1 and WDR4 is shown in Figure 2B. The expression distribution of these lncRNAs is shown in Figure 2C. A univariate Cox analysis verified the associations between hub lncRNAs and prognosis (Figure 2D). We further detected correlations between the levels of these lncRNAs (Figure 2E). We found significantly higher expression levels of AC099811.3, AC125807.2, AC018529.2, and AC015911.3 in CM samples than in normal samples (*p* < 0.05; Figure 3C–3F), whereas MCCC1-AS1 and AATBC were lower expressed in CM (*p* < 0.05; Figure 3A and 3B). Patients with CM in TCGA cohort were subsequently divided into low- and high-risk groups based on the median risk scores (Figure 4A and 4B). t-SNE and PCA analyses indicated that CM patients in the two risk subgroups were clearly separated (Figure 4C and 4D).

### Associations between clinical characteristics and risk scores in CM

**Figure 5:**
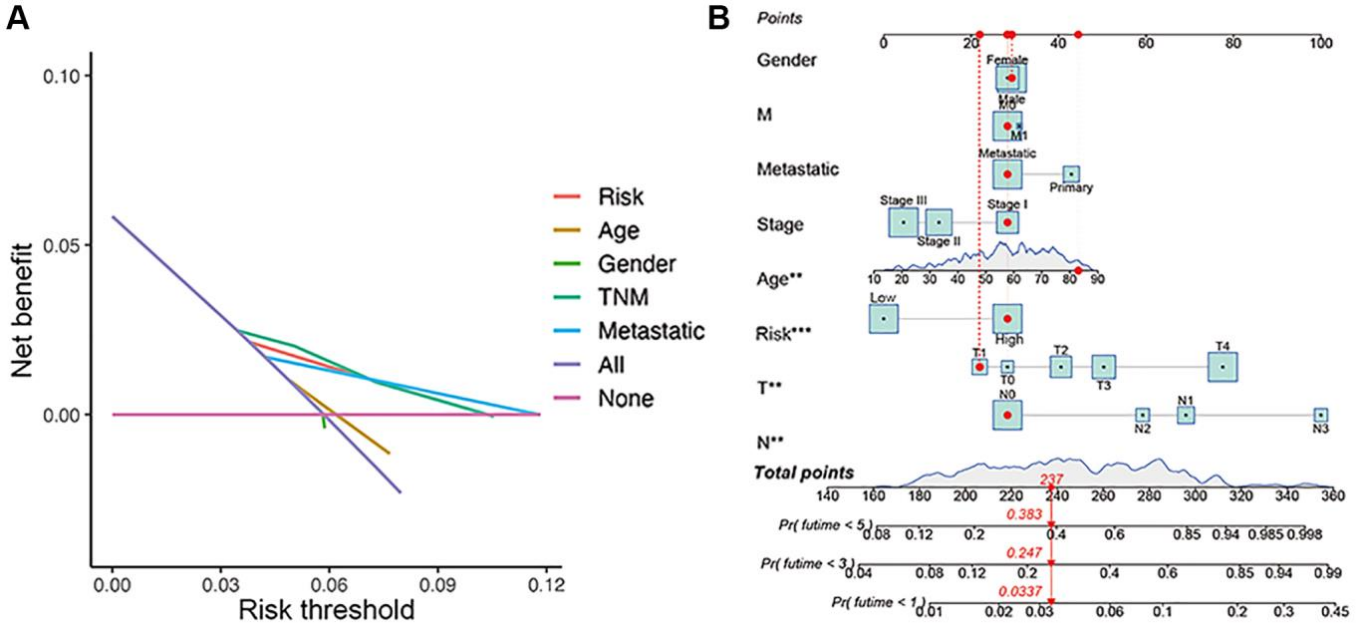
**Construction of nomogram.** (**A**) Decision curve analysis of risk signature and other clinicopathological features. (**B**) Nomogram for predicting CM 1-, 3-, and 5-year overall survival in TCGA cohort. The red dashed line represented a sample of CM patient's death probability by year 1, 3, and 5.

The overall survival was lower in the high-risk CM group than in the low-risk group in TCGA cohort (Figure 4E). A receiver operating characteristic (ROC) curve analysis indicated that the risk signature had moderate predictive accuracy at 1 (ROC = 0.691), 2 (ROC = 0.703), and 3 (ROC = 0.677) years (Figure 4F). A decision curve analysis and ROC analysis proved that the signature has greater accuracy than all other traditional clinicopathological features (Figure 4G and 5A), revealing that our risk signature is a sensitive and specific predictor of overall survival in CM.

Multivariate and univariate Cox regression analyses revealed that the newly identified risk signature is an independent prognostic factor for CM patients (Figure 4H and 4I). Interestingly, a heatmap of clinical features and risk subgroups showed that our gene signature was significantly associated with tumor T stage and the metastatic statue (Figure 4L). CM patients with higher T stage had significantly greater risk scores than those with low T stage (Figure 4J). Meanwhile, CM patients diagnosed with primary melanoma also showed significantly higher risk scores than patients with metastatic melanoma (*p* < 0.05; Figure 4K). Finally, the risk signature was used to construct a nomogram to predict CM patient outcomes (Figure 5B). Overall, our constructed risk signature was clearly associated with the development of CM and might be a valuable tool for the clinical management of patients.

### GSEA

**Figure 6:**
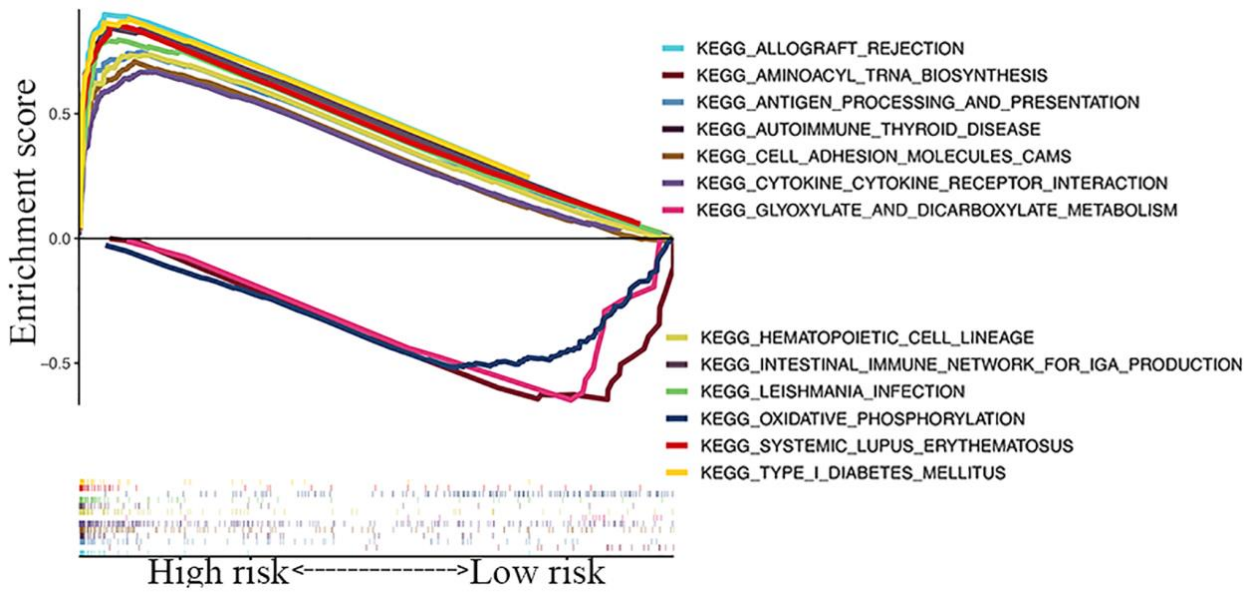
GSEA of top 13 enriched pathways in risk signature.

A KEGG pathway enrichment analysis discovered that the identified lncRNA signature was significantly enriched in 47 pathways (FDR <0.05) (Supplementary Table 5), including oxidative phosphorylation, aminoacyl tRNA biosynthesis, glyoxylate and dicarboxylate metabolism, systemic lupus erythematosus, cytokine-cytokine receptor interaction, and type 1 diabetes mellitus (Figure 6). Several immune-related pathways, such as intestinal immune network, natural killer cell-mediated cytotoxicity, and primary immunodeficiency pathways were also enriched.

### Associations with immunity, tumor stemness, and M6A-related genes

**Figure 7:**
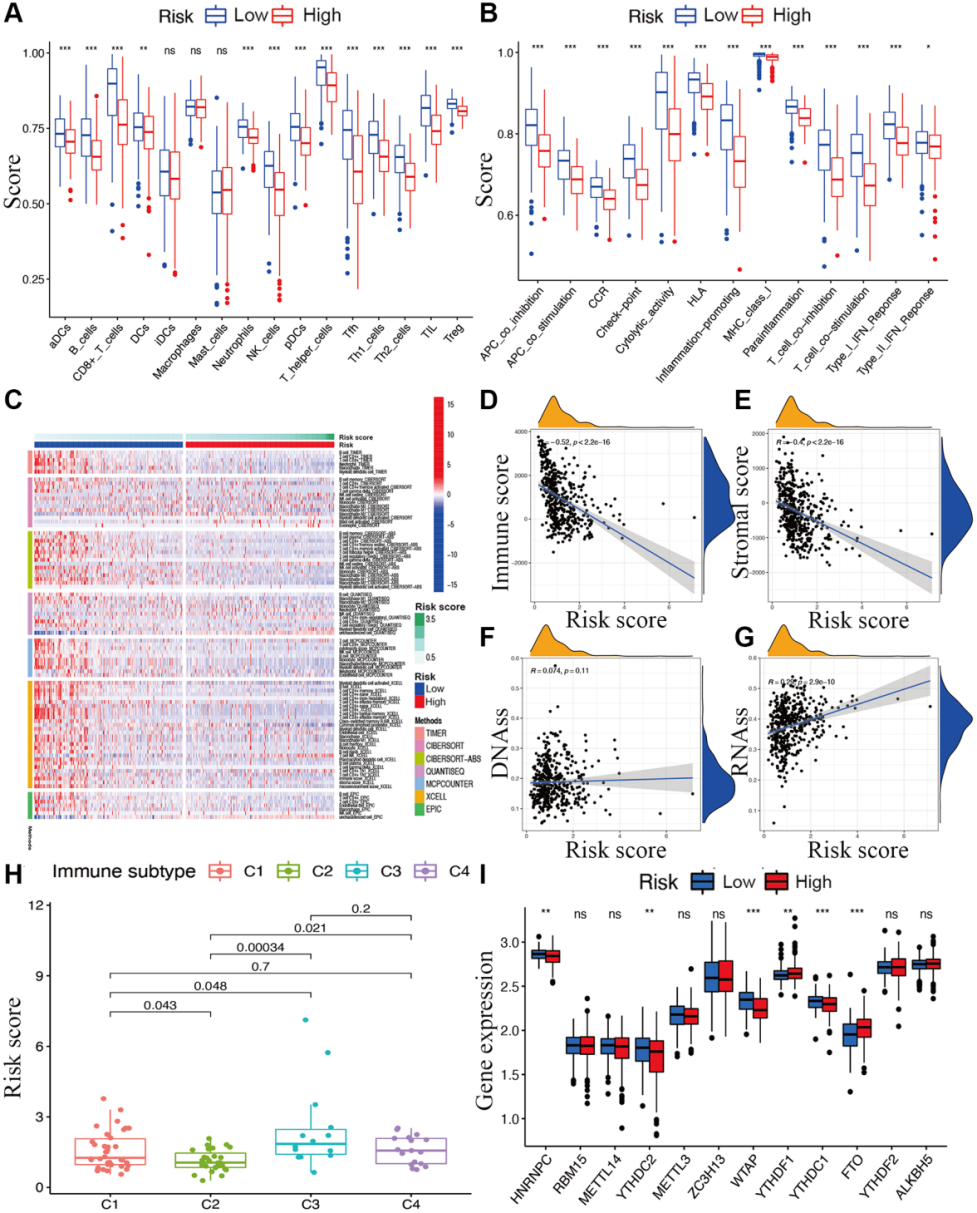
**Potential role of risk signature in CM immune status, tumor stemness, and m6A-related genes.** Boxplots of scores of immune cells (**A**) and immune-associated functions (**B**) in risk subgroups. (**C**) Heatmap for immune responses based on EPIC, XCELL, MCP counter, QUANTISEQ, CIBERSORT, and TIMER among two risk subgroups. Associations between risk signature and immune scores (**D**), stromal scores (**E**), DNAss (**F**), RNAss (**G**), immune infiltration subtypes (**H**), and m6A-related genes (**I**).

While exploring the relationship between risk signature and cancer immunity, the results displayed that the proportion of most immune cell subpopulations, levels of components of related pathways, and functions were significantly reduced in the high-risk subgroup compared with the low-risk subgroup (*p* < 0.05; Figure 7A and 7B). Only the scores for iDCs, macrophages, and mast cells did not differ significantly between the two subgroups (*p* > 0.05). Similar results were obtained using EPIC, XCELL, MCP counter, QUANTISEQ, CIBERSORT, and TIMER analysis (Figure 7C). Furthermore, immune infiltrates corresponding to tumor suppression and promotion [17], namely C1 (wound healing), C2 (INF-g dominant), C3 (inflammatory), and C4 (lymphocyte-depleted), were evaluated to understand the connection between immune components and the risk signature. The calculated risk score was significantly higher for the C3 subtype and lower for the C2 subtype than for other subtypes (Figure 7H).

The tumor stemness (including the RNA stemness score and DNA methylation pattern), immune microenvironment (including immune and stromal scores), and m6A-related genes are all key regulators of tumor progression. The constructed lncRNA signature was significantly negatively correlated with the immune and stromal scores (Figure 7D and 7E) but positively correlated with RNA methylation patterns (RNAss; Figure 7F and 7G). Expression levels of the m6A-related genes *FTO* and *YTHDF1* were significantly lower and *HNRNPC*, *YTHDC2*, *YTHDC1*, and *WTAP* were higher in the low-risk subgroup than in another subgroup (Figure 7I).

**Figure 8:**
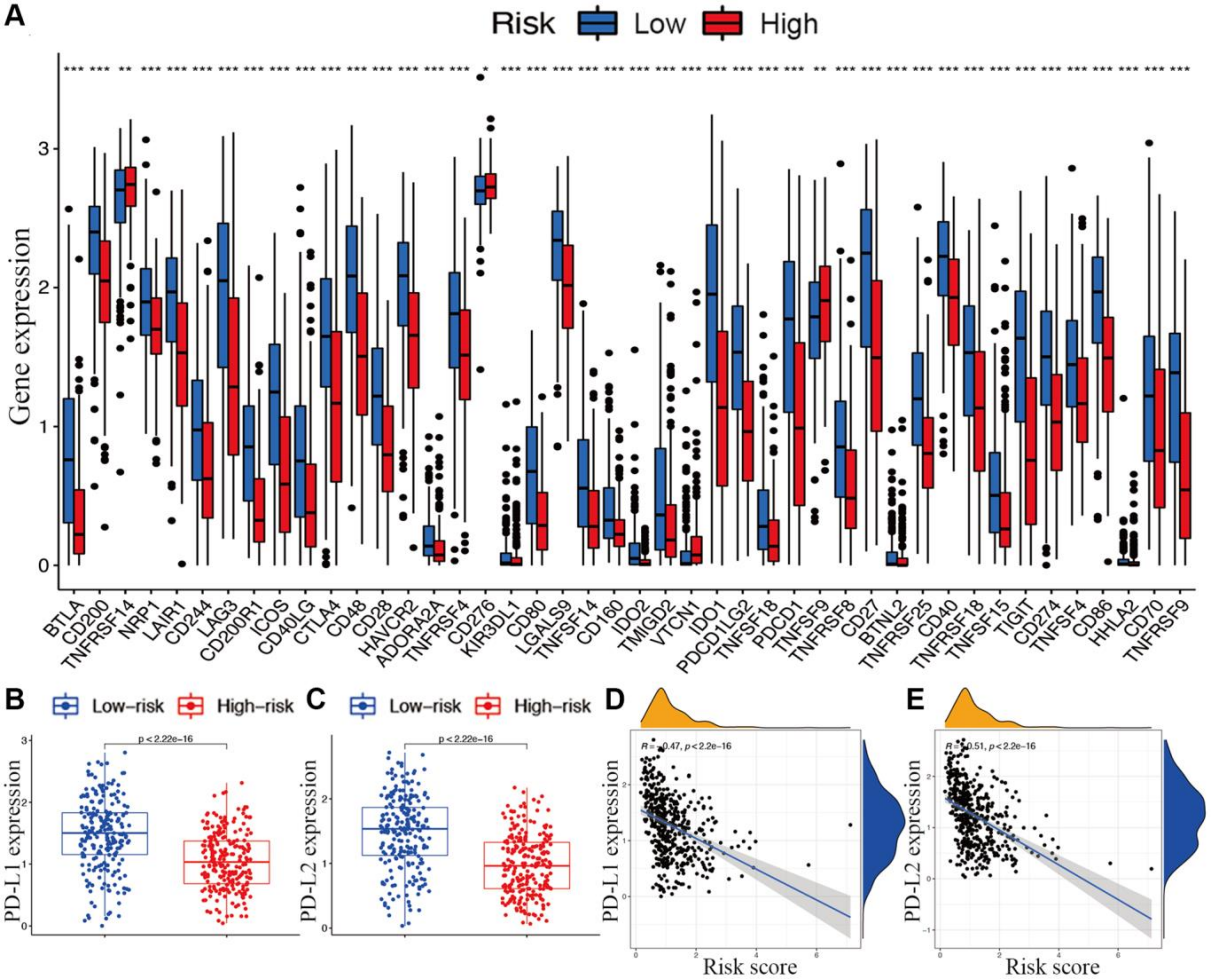
**Associations between risk signature and immune checkpoints.** (**A**) Expression of immune checkpoints among two risk subgroups in CM patients. Expression levels of genes PD-L1 (**B**) and PD-L2 (**C**) in risk subgroups. Correlation analysis between risk score, PD-L1 (**D**), and PD-L2 (**E**).

With respect to immune checkpoints, the levels of most identified immune-related points were lower expressed in the high-risk subgroup, except for TNFRSF14, *CD276*, and *TNFSF9* (Figure 8A). Moreover, considering the roles of the immune checkpoint protein *PD-L1* and *PD-L2* in immune evasion, the relationship between these loci and the lncRNA signature was analyzed comprehensively. The gene expression levels of *PD-L1* and *PD-L2* were both significantly higher in the low-risk subgroup than in the high-risk subgroup (Figure 8B and 8C). Meanwhile, the expression of *PD-L1* and *PD-L2* were significantly negatively correlated with the risk score (Figure 8D and 8E).

## DISCUSSION

Although next-generation sequencing technology has resulted in the discovery of various biomarkers for melanoma, there is still a need for novel markers that are more closely associated with early detection and prognosis in CM. M7G, a novel uncharacterized cell death mechanism, which is significantly correlated with human cell death, has potentially treatment value in melanoma [18]. However, its role in melanoma is poorly understand, and a m7G-associated lncRNA signature has not been reported. Our newly identified lncRNA signature showed high predictive accuracy for overall survival in CM. Meanwhile, our lncRNA signature was also associated with the immune status, tumor microenvironment, immune components, m6A-related genes, and tumor stemness, presenting an advantage over other risk signatures.

In the present study, m7G methylation complexes METTL1 and WDR4 were systematically analyzed to identify lncRNAs associated with CM overall survival. Next, 6 hub lncRNAs, including MCCC1-AS1, AC099811.3, AC125807.2, AC018529.2, AC015911.3, AATBC, were used to construct a novel prognostic signature for CM. The prognostic value for CM was verified by various approaches. The identified signature was significantly correlated with metastasis and T stage. Regarding the prognostic value of risk signature in CM metastatic, i.e., the emergence of higher risk scores in primary CM, we consider that this was mainly due to the minor number of primary CM samples in TCGA cohort. Factually, there were only 104 patients diagnosed with primary CM, but 366 patients corresponded with metastatic cancer. Meanwhile, as reported by previous literature, TCGA-CM patients with primary cancer had significantly worse overall survival [19], which is consistent with the findings of the relationship between CM overall survival, risk score, and metastatic status in this study. The American Joint Committee on Cancer (AJCC) staging system is a widely used clinicopathological parameter for tumor evaluations [20]. Compared with the TNM stage, irrespective of whether T, N, and M stages are considered separately or together, the constructed risk signature not only showed a higher accuracy for the prediction of prognosis but also could be used to predict CM growth and metastatic potential. A nomogram analysis revealed the effectiveness of this lncRNA signature for predicting the outcomes of CM patients.

Based on a GSEA, the risk signature was enriched in several immune-related pathways, such as intestinal immune network, natural killer cell-mediated cytotoxicity, and primary immunodeficiency pathways. Meanwhile, the signature was also connected with several immune-associated diseases, such as systemic lupus erythematosus and autoimmune thyroid disease. Thus, the prognostic value of the lncRNA signature might be attributed to its association with immune processes. Interestingly, nearly all immune cells showing reduced infiltration and immune functions were inhibited in the high-risk subgroup. Given the critical roles of these immune cells in stimulating anti-tumor immunity [21], it is reasonable to conclude that the degree of anti-tumor immunity in patients with CM in the high-risk subgroup is substantially reduced. In addition, the ESTIMATE algorithm demonstrated that the stromal cell and immune cell scores were both negatively correlated with the risk score, confirming that immune cell infiltration was poor in the high-risk subgroup. The analysis concerning the association between immune components and CM confirmed that C2 and C3 were both significantly connected with declined risk scores. Considering the predictive value of the risk signature in overall survival, C2 and C3 might be protective factors in CM.

Cancer immunotherapies targeting immune checkpoints have improved outcomes in various cancers [22]. PD-L1 and PD-L2 are key regulators of immune responses [23]. Positive PD-L1 expression is correlated with better clinical outcomes in melanoma. Clinical trials have demonstrated that PD-1/PD-L1 pathway-targeted monoclonal antibodies result in impressive outcomes in CM by preventing the inhibition of the PD-L1 pathway and enhancing the function of T cells [24, 25]. In this study, the significantly differential expression of PD-L1 and PD-L2 in the two risk groups were also verified as well as the fact that they are both negatively correlated with the risk score. Levels of nearly all immune checkpoints were significantly higher in the low-risk subgroup than in the high-risk subgroup, suggesting that immune responses were dramatically altered in high-risk group. The identified lncRNA signature could predict the expression of immune checkpoints in CM and potentially guide the implementation of immunotherapy. However, the specific connections between m7G lncRNAs and immune-related genes still warrant further explorations.

Cancer stem cell-like cells (CSCs) promote tumor growth owing to their self-renewal and invasion abilities. CSCs are also the main determinant of chemotherapy drug resistance [26, 27]. In the present study, our lncRNA signature was positively correlated with the stem cell score, confirming that our newly constructed gene signature was a risk factor for CM. Similar to m7G, N6-methyladenosine (m6A) is the other most abundant methylation form in human cells, occurring mainly on the adenine of the RRACH sequence. Meanwhile, m6A was identified to be connected with a variety of cancers and human physiologies [28], especially tumor immunity [6, 7]. Thus, it is valuable to explore the relationship between m7G and m6A. In this study, our m7G-related lncRNA signature could effectively predict the expression levels of the m6A-related genes *FTO*, *YTHDF1*, *HNRNPC*, *YTHDC2*, *YTHDC1*, and *WTAP*, although the specific mechanisms underlying these relationships need further exploration.

Despite the prognostic value of the current risk signature, this study also has some limitations. First, the results from our present retrospective study need further confirmation by prospective studies. Second, more experimental assays are needed to verify and validate the conclusions obtained from bioinformatics analyses. In the future, functional studies should be performed to gain mechanistic insights into m7G-associated lncRNAs and their role in CM development.

In the present study, a novel prognostic risk signature consisting of 6 hub m7G-associated lncRNAs was constructed and presented high predictive accuracy. This gene signature was valuable to predict parameters related to immune components, immune functions, immune cell infiltration, tumor microenvironment, stemness, and m6A-related genes in CM patients. To the best of our knowledge, this is the first m7G-related lncRNA signature for cancer. These results also provided a novel basis for understanding the specific effects of m7G-related lncRNA in CM. Therefore, this study comprehends a significant contribution to the literature and can contribute to improvements in the outcomes and individualized treatments for CM patients.

## MATERIALS AND METHODS

### Raw data acquisition

RNA and lncRNA sequencing data were collected for 471 CM tissues and one normal skin sample from The Cancer Genome Atlas (TCGA) database on June 30, 2020 (https://portal.gdc.cancer.gov). The transcriptomic data for 812 normal skin samples were obtained from the Genotype-Tissue Expression database (GTEx; https://gtexportal.org/home/datasets). Log2-transformation and normalization were performed using the “sva” package in R to remove batch effects [29, 30]. Specific m7G methylation complexes METTL1 and WDR4 (Supplementary Table 6) were applied for further analysis.

### Prognostic lncRNA signature construction

After assessing the association between m7G-associated lncRNAs and CM by a Pearson correlation analysis (|R^2^| > 0.2, *p* < 0.01), differentially expressed m7G-associated lncRNAs were identified using the “limma” package. lncRNAs with |log2 fold change| > 1 and false discovery rate (FDR) <0.05 between normal and tumor tissues were regarded as candidate lncRNAs. Then, univariate Cox regression analyses were performed to identify prognostic m7G-associated lncRNAs among all lncRNAs in CM by using the “survival” package with a cutoff of *p* < 0.001. The overlap between the differentially expressed lncRNAs and prognosis-related lncRNAs was determined as candidate m7G-related lncRNAs, as visualized by a Venn diagram using the “VennDiagram” package in R. Thereafter, candidate m7G-related lncRNAs were integrated into a Lasso penalized Cox regression analysis to identify hub lncRNAs and to generate a lncRNA risk model. Next, patients with CM were categorized into low- and high-risk groups using the median risk score as a threshold. The risk score was calculated as follows:


risk score=∑exp lncRNAi×βi

where exp lncRNAi is the relative expression of hub m7G-related lncRNA i, and β is the regression coefficient [31].

### Predictive value of the lncRNA signature

To explore the distribution of risk subgroups, t-SNE and PCA analyses of the constructed signature were performed using the “Rtsne” and “ggplot2” packages in R. The “survival” package was further applied to compare overall survival between the two risk subgroups according to risk scores. To verify the predictive accuracy of the signature, the “timeROC” package was also applied for both the lncRNA signature and traditional clinical features. Finally, univariate and multivariate Cox regression analyses were performed to evaluate the relationship between the risk score and clinical characteristics. To translate the prognostic value of risk signature into CM clinical use, a nomogram which included the risk statue and the CM clinical characteristics gender, M stage, metastatic, AJCC stage, age, T stage, and N stage was constructed using the “rms” package. Bootstraps with 1000 resamples were applied to internally validate the constructed nomogram. Meanwhile, the decision curve analysis was further applied to detect the clinical usefulness of the risk nomogram by quantifying the net benefits at different threshold probabilities.

### Gene set enrichment analysis (GSEA)

For the hub m7G-associated lncRNAs, GSEA 4.1 was used for a Kyoto Encyclopedia of Genes and Genomes (KEGG) enrichment analysis of the two risk subgroups. Statistical significance was defined as FDR <0.05.

### Immune, stem cell-like features, and M6A correlation analysis

A Spearman correlation analysis was performed to test the relationship between the risk score and stromal and immune scores. Two-way ANOVA was used to evaluate the connection between the immune infiltration subtype and risk score. A single-sample gene set enrichment analysis (ssGSEA) was used to compare immune cell infiltration in the two risk subgroups and to test immune functions. Potential immune checkpoints retrieved from a previous study were used to explore the connection between immune-related genes and risk signatures [32]. Next, correlations between the risk signature and two key immune regulators, including PD-L1 and PD-L2, were evaluated. Spearman correlation analyses were used to measure the relationship among the risk score, tumor stemness, and m6A-related genes.

### Availability of data and materials

The datasets used and/or analyzed during the current study are available from the corresponding author on reasonable request.

## Supplementary Materials

Supplementary Table 1

Supplementary Table 2

Supplementary Tables
