# Supplementary material for: Identification of m7G-associated lncRNA prognostic signature for predicting the immune status in cutaneous melanoma
Source: Aging (Albany NY). 2022 Jun 29;14(12):5233–49. doi: 10.18632/aging.204151 (PMC9271298; doi:10.18632/aging.204151)
Supplement: Supplementary Tables [file aging-14-204151-s003.pdf]

SUPPLEMENTARY TABLES

Supplementary Table 3. Identification differentially expressed lncRNAs by differential expression analysis.

lncRNA	HR	HR.95L	HR.95H	*P*-value
AC011481.1	1.56650331	1.22383658	2.00511462	0.00036564
HCG27	0.45898288	0.30133005	0.69911809	0.00028663
LINC02384	0.46031389	0.31577208	0.67101841	5.47E-05
AF127936.1	0.54100385	0.40795747	0.71744038	1.99E-05
TFAP2A-AS1	1.24478017	1.11948166	1.38410277	5.23E-05
LINC00520	1.12091787	1.05120729	1.1952513	0.00049332
LINC00665	1.35662711	1.14900111	1.60177141	0.00031969
AC025154.2	1.52306951	1.22108543	1.89973665	0.00019036
AC036108.3	0.54390733	0.41462378	0.71350269	1.09E-05
MCCC1-AS1	0.42908919	0.26328605	0.69930607	0.00068573
PCED1B-AS1	0.776997	0.68528489	0.880983	8.24E-05
AC093726.1	0.67253079	0.56361832	0.80248928	1.08E-05
AC004687.1	0.75117625	0.64712113	0.87196311	0.00016931
AC099811.3	1.50161761	1.22172194	1.8456372	0.00011213
AL161452.1	2.20773316	1.46747696	3.32140528	0.00014438
AL353622.1	0.47680549	0.33518839	0.67825584	3.80E-05
AC099343.2	0.41728228	0.27787955	0.62661863	2.52E-05
PCOTH	1.53996976	1.23480128	1.92055749	0.00012727
AC015911.8	0.59045352	0.46333654	0.7524452	2.05E-05
AC125807.2	1.48481645	1.22921973	1.79356046	4.11E-05
AC097468.3	1.38247271	1.14462641	1.66974199	0.00077305
STARD7-AS1	2.07841144	1.51211125	2.85679648	6.55E-06
LINC00324	0.59631703	0.46349005	0.76720957	5.79E-05
AC018755.4	0.71685755	0.59813291	0.85914808	0.0003142
AC078883.1	0.37979171	0.24985642	0.57729852	5.86E-06
EBLN3P	0.64736264	0.51606489	0.81206529	0.00016993
AC018529.2	1.35663209	1.16386626	1.58132483	9.59E-05
AC006504.5	1.69973509	1.27426221	2.26727226	0.00030764
AC005332.7	0.34031825	0.18045058	0.64181842	0.00086865
AC100791.3	1.35318896	1.16030692	1.57813448	0.00011573
AC007728.2	0.48477945	0.33518912	0.70112988	0.00012011
AL133371.2	0.64919108	0.52586535	0.8014391	5.84E-05
HEXA-AS1	2.52358589	1.57470971	4.04422841	0.00011955
AC131009.3	1.48837894	1.22352146	1.81057049	6.96E-05
B4GALT1-AS1	1.76012131	1.33876495	2.3140933	5.13E-05
NRAV	1.67851127	1.2925116	2.17978706	0.00010257
AC008915.2	1.48038045	1.1873322	1.84575665	0.00049101
MIR155HG	0.69984592	0.60317438	0.81201113	2.53E-06

AC015911.3	0.57448594	0.46000983	0.7174501	1.02E-06
AC106782.5	0.42589628	0.2586919	0.70117251	0.00079202
AC135050.1	2.63129805	1.48021439	4.67751799	0.00098033
AC083799.1	0.64445772	0.52046757	0.79798583	5.58E-05
AL683807.1	0.45475331	0.32216857	0.64190177	7.44E-06
AC012645.3	0.56520315	0.41812708	0.76401316	0.00020698
AC020659.1	0.25231656	0.11694113	0.54440767	0.00044861
RNF216P1	1.6269999	1.25101655	2.11598214	0.00028304
AC108134.3	0.46827351	0.32987919	0.66472843	2.19E-05
PSMB8-AS1	0.77344215	0.68765064	0.86993703	1.85E-05
LINC02328	0.63121668	0.48520796	0.82116234	0.0006082
AC015912.3	1.26871696	1.10703255	1.45401571	0.00062186
AC116025.2	1.66972782	1.30400058	2.13802895	4.82E-05
ATXN2-AS	1.69980393	1.24199123	2.32637183	0.00092115
RERE-AS1	0.33897162	0.18059819	0.63622875	0.00075833
WAC-AS1	0.6536467	0.51110032	0.83594939	0.00070507
BHLHE40-AS1	0.43506839	0.29620948	0.63902244	2.20E-05
AC011445.1	1.55273449	1.22025397	1.97580543	0.00034476
LINC00539	0.35787541	0.21659892	0.59129938	6.05E-05
AL138966.2	2.13385407	1.45016603	3.13987026	0.00012007
HCP5	0.8229855	0.75933249	0.89197438	2.10E-06
SNAI3-AS1	1.75905663	1.3008702	2.37862333	0.00024399
AC004471.1	1.5841252	1.21202264	2.07046681	0.00075826
AC067852.2	1.58849867	1.24722871	2.02314781	0.00017667
JARID2-AS1	1.93790088	1.37708625	2.72710574	0.00014727
AC005083.1	0.58698914	0.43235959	0.79692056	0.00063754
MIAT	0.59483631	0.4688713	0.75464256	1.88E-05
PAXIP1-AS2	0.66879761	0.53180774	0.8410751	0.00058175
AC018553.1	1.51632131	1.29969373	1.76905549	1.21E-07
VIM-AS1	0.55750289	0.41981824	0.74034294	5.41E-05
AC005498.2	2.45169552	1.5255476	3.94010054	0.00021156
AC004221.1	1.79201268	1.26706071	2.53445587	0.00097269
AC243960.1	0.67161311	0.56538005	0.79780701	5.86E-06
LINC01502	1.13815282	1.05799387	1.224385	0.00051489
AC009570.1	1.47022968	1.18328829	1.82675289	0.00050293
LINC01679	1.21800306	1.08776918	1.36382927	0.00063063
HLA-DQB1-AS1	0.68880264	0.59565026	0.79652291	4.94E-07
AATBC	1.46255189	1.26319705	1.69336845	3.68E-07
ITGB2-AS1	0.70824898	0.59496363	0.84310469	0.00010485
AL365361.1	0.65586828	0.54658686	0.78699881	5.74E-06
AC025171.5	0.29584811	0.1593311	0.54933473	0.00011471
AC008622.2	2.2117542	1.39965942	3.49503355	0.00067333

AL034346.1	1.61660375	1.31068148	1.99393042	7.20E-06
AL022067.1	0.39103755	0.22906745	0.66753425	0.00057913

Supplementary Table 4. Identification prognostic lncRNAs by univariate Cox regression.

LncRNA	Coef	HR	HR.95L	HR.95H	*P*-value
MCCC1-AS1	−0.4592578	0.63175236	0.38828774	1.02787446	0.06442031
AC099811.3	0.19646052	1.21708727	0.97699268	1.51618478	0.07971361
AC125807.2	0.17746946	1.19419158	0.97918359	1.45641079	0.07972881
AC018529.2	0.1951604	1.21550594	1.03158172	1.43222262	0.01973187
AC015911.3	−0.4228181	0.65519783	0.52262596	0.82139854	0.00024667
AATBC	0.20004932	1.221463	1.03951296	1.43526048	0.0150633

Supplementary Table 5. Identification candidate prognostic lncRNAs by a Lasso penalized Cox regression analysis.

Name	Size	FDR *q*-value
KEGG_OXIDATIVE_PHOSPHORYLATION	131	0.00152385
KEGG_AMINOACYL_TRNA_BIOSYNTHESIS	22	0.00825053
KEGG_GLYOXYLATE_AND_DICARBOXYLATE_METABOLISM	16	0.01605843
KEGG_SYSTEMIC_LUPUS_ERYTHEMATOSUS	56	0
KEGG_CYTOKINE_CYTOKINE_RECEPTOR_INTERACTION	263	0
KEGG_TYPE_I_DIABETES_MELLITUS	41	0
KEGG_AUTOIMMUNE_THYROID_DISEASE	50	0
KEGG_CELL_ADHESION_MOLECULES_CAMS	131	0
KEGG_LEISHMANIA_INFECTION	69	0
KEGG_INTESTINAL_IMMUNE_NETWORK_FOR_IGA_PRODUCTION	45	0
KEGG_ALLOGRAFT_REJECTION	35	0
KEGG_HEMATOPOIETIC_CELL_LINEAGE	85	0
KEGG_ANTIGEN_PROCESSING_AND_PRESENTATION	81	0
KEGG_GRAFT_VERSUS_HOST_DISEASE	37	0
KEGG_NATURAL_KILLER_CELL_MEDIATED_CYTOTOXICITY	132	0
KEGG_CHEMOKINE_SIGNALING_PATHWAY	188	0
KEGG_PRIMARY_IMMUNODEFICIENCY	35	0
KEGG_ASTHMA	28	0
KEGG_VIRAL_MYOCARDITIS	68	0
KEGG_JAK_STAT_SIGNALING_PATHWAY	155	0
KEGG_T_CELL_RECEPTOR_SIGNALING_PATHWAY	108	0
KEGG_TOLL_LIKE_RECEPTOR_SIGNALING_PATHWAY	102	0
KEGG_COMPLEMENT_AND_COAGULATION_CASCADES	69	0
KEGG_NOD_LIKE_RECEPTOR_SIGNALING_PATHWAY	62	0
KEGG_B_CELL_RECEPTOR_SIGNALING_PATHWAY	75	0
KEGG_LEUKOCYTE_TRANSENDOTHELIAL_MIGRATION	116	0
KEGG_APOPTOSIS	87	0

KEGG_ECM_RECEPTOR_INTERACTION	84	4.26E-05
KEGG_RIG_I_LIKE_RECEPTOR_SIGNALING_PATHWAY	70	1.36E-04
KEGG_CYTOSOLIC_DNA_SENSING_PATHWAY	54	2.71E-04
KEGG_PRION_DISEASES	35	3.98E-04
KEGG_FOCAL_ADHESION	199	5.15E-04
KEGG_FC_GAMMA_R_MEDIATED_PHAGOCYTOSIS	96	9.06E-04
KEGG_REGULATION_OF_ACTIN_CYTOSKELETON	213	9.14E-04
KEGG_HYPERTROPHIC_CARDIOMYOPATHY_HCM	82	0.00221456
KEGG_DILATED_CARDIOMYOPATHY	89	0.00214745
KEGG_TGF_BETA_SIGNALING_PATHWAY	84	0.00278056
KEGG_FC_EPSILON_RI_SIGNALING_PATHWAY	79	0.00408717
KEGG_MAPK_SIGNALING_PATHWAY	266	0.00516064
KEGG_LONG_TERM_POTENTIATION	70	0.00627491
KEGG_ARRHYTHMOGENIC_RIGHT_VENTRICULAR_CARDIOMYOPATHY_ARVC	74	0.01377414
KEGG_PATHWAYS_IN_CANCER	324	0.01738811
KEGG_NEUROACTIVE_LIGAND_RECEPTOR_INTERACTION	271	0.02168642
KEGG_VASCULAR_SMOOTH_MUSCLE_CONTRACTION	114	0.0238143
KEGG_P53_SIGNALING_PATHWAY	68	0.03537415
KEGG_SMALL_CELL_LUNG_CANCER	84	0.03872098
KEGG_UBIQUITIN_MEDIATED_PROTEOLYSIS	134	0.04711841

Supplementary Table 6. KEGG pathway enrichment analysis.

Gene symbol	Description
METTL1	Methyltransferase 1, TRNA Methylguanosine
WDR4	WD Repeat Domain 4
